# ‘With a Little Help from My Friends’: Emotional Intelligence, Social Support, and Distress during the COVID-19 Outbreak

**DOI:** 10.3390/ijerph20032515

**Published:** 2023-01-31

**Authors:** Dorota Kornas-Biela, Klaudia Martynowska, Leehu Zysberg

**Affiliations:** 1 Institute of Pedagogy, The John Paul Catholic University of Lublin, 20-950 Lublin, Poland; 2 The Graduate School, Gordon College of Education, Haifa 3465415, Israel

**Keywords:** emotional intelligence, COVID-19, depression, stress, anxiety, social support, students

## Abstract

The COVID-19 pandemic presented a global existential social and health challenge, with individuals suffering mentally and psychologically. College and university students are young adults, typically away from their natural support systems; with pandemic-imposed measures such as isolation, they may have been at higher risk of experiencing negative psychological outcomes. The study tested a model in which social support mediated the association between emotional intelligence (EI) and a latent factor representing general mental distress at the height of the COVID-19 crisis in Poland. One hundred and fifty-nine young adults filled in measures of trait EI, psychological and instrumental social support, three distress measures (depression, anxiety, and stress), and demographics. The results supported a model in which psychological social support (but not instrumental social support) mediated the association between trait EI and a factor representing all three distress measures. The results shed light on how individual and social resources work together to help maintain psychological integrity in times of crisis. They add to recent results on the differential effects of psychological-emotional and instrumental social support on distress and well-being.

## 1. Introduction

The COVID-19 pandemic took the world by surprise, presenting a global existential social and health challenge to most nations and societies and, of course, to individuals. While the WHO has defined the pandemic as a serious health threat [1], its main detrimental outcomes are social, economic, and psychological rather than physical [2]. Consequently, mental health and well-being have become major topics of discussion [3,4,5]. Ample evidence shows individuals and groups have paid a heavy emotional and psychological toll, with distress and other negative psychological outcomes commonly reported (e.g., [1]).

The first studies on COVID-19 were mainly concerned with medical and epidemiological issues, but quickly turned to problems associated with social distancing and isolation [6]. Studies found the limitations set to contain the pandemic were associated with symptoms of depression, generalized anxiety disorder, intrusive thoughts, insomnia, and acute stress [7]. Other studies found the responses to the pandemic and the measures taken to counter it led to acute stress, anxiety, depression, loneliness, insomnia, alcohol and drug abuse, self-harm or suicidal behavior, fear (of meeting other people, of death, of isolation, of lacking essential items), and neurotic or obsessive-compulsive disorders [8,9,10,11]. Additional studies looked specifically at the detrimental effects of quarantine: stressors included quarantine duration, infection fears, frustration, boredom, inadequate information and supplies, financial loss, and stigma [12]. Repeated media coverage of the pandemic also had far-reaching deleterious effects on physical and mental health [13,14]. People who spent more time thinking about the outbreak (≥3 h) were more likely to develop anxiety symptoms [15]. In fact, the literature suggests that pandemic-related stress, anxiety, and depression appeared in about every third person [16].

Studies of COVID-19 related consequences have focused on at-risk populations and health professionals, but college and university students represent an interesting population in the context of the pandemic. Students are young adults, typically away from their natural support systems, and as campuses closed and students found themselves in isolation, evidence began to suggest this target population was at high risk of experiencing negative psychological outcomes [17]. Studies documented the negative psychological effects of the pandemic in college and university students even in the first months of the pandemic [14,18,19,20,21,22]. The results of studies in different countries suggest students displayed more psychological distress and suffered more often from mental health disorders than their peers. Common problems included feelings of being overwhelmed and emotionally exhausted, fatigue, sleep disturbances, restlessness, anxiety, depressive states, low levels of optimism, lowered self-esteem, and reduced self-efficacy [23]. In one study, more than half of the university students sampled suffered from stress, anxiety, and fear, and many needed support from formal sources such as university staff in addition to family support [24].

A significant predictor of stress reactions in students is gender. Female college students bear a disproportionate burden of mental health problems worldwide, with higher levels of stress and related symptoms (anxiety and depression) than male students [25]. This pattern was confirmed during the pandemic. Students all over the world showed a very high burden of mental health problems, but women had higher levels of stress [26], anxiety, and depression than men [11,18,27]. In China, anxiety was the most common and serious problem for students, especially female students, while male students showed a tendency to depression [28].

A concept representing a range of negative psychological outcomes for both individuals and groups is psychological distress. While the concept is defined differently in various settings and contexts, it represents a state of suffering, typically marked by stress, anxiety, and depression [29]. Psychological distress can be placed on a continuum—ranging from mild inconvenience to overcoming debilitating experience [30]. Evidence from the health sciences and the behavioral sciences points to the importance of the concept in any diagnostic or care-related context as a subjective factor associated with a wide range of behaviors and outcomes, especially in times of challenge and crisis [31].

Studies examining the dynamics of distress versus well-being in the face of an immediate threat suggest there are two major types of resources: personal and social [32,33,34]. Personal resources may include personal characteristics, abilities, and skills that can be used to cope with stress and threat more effectively, from problem-solving skills and knowledge, to optimism and sense of humor—all associated with more effective coping and better outcomes at the aftermath of coping with stressful events [33]. One of the most popular individual resources mentioned in the literature within the context of psychological stress and threat is emotional intelligence (EI). Social resources pertain to the availability and nature of interpersonal, formal, and informal networks and dynamics that provide practical or psychological coping capacity to individuals and groups. Evidence suggests that chief among these resources is social support [35]. While studies have shown evidence of associations between emotional intelligence and other individual resources, as well as social support, with indicators of well-being on one hand and distress on the other hand, the potential associations and interrelations between individuals and social resources in the face of threat are yet to be thoroughly studied.

The COVID-19 pandemic presented an opportunity to test the potential dynamics between different types of resources in the process of coping with threat and the potential outcomes. We built on this to examine the resources associated with the experience of distress among young adult students during the COVID-19 pandemic. We tested a multi-tiered model in which individual level and inter-personal level resources work together to determine distress reactions to the COVID-19 threat. We selected college students because this population is under-represented in studies in this area.

The main findings suggested the associations between EI and distress were partially mediated by social support, but this was mainly the emotional component of support, not than the instrumental one.

## 2. Literature Review

### 2.1. Individual Level Resources

Individual resources are personal characteristics that may buffer stress reactions and help people maintain a sense of purpose, control, and well-being when coping with threats. Theoretical and empirical studies identify factors such as self-esteem, self-efficacy, sense of coherence, hope, and personality predispositions such as a sense of humor and optimism as major individual resources [33,35] associated with effective coping and reduced anxiety and psychological distress in the face of challenges.

A relatively recent addition to this group of resources is the concept of emotional intelligence (EI). The concept represents an amalgam of personal characteristics that enable individuals to identify and process emotions effectively, manage conflict in the self and others, and harness emotional reactions to promote effective coping behavior [36]. We adopted a personality approach to EI, conceptualizing this resource as a personality predisposition amalgam located along the rigidity-flexibility axis of personality [37].

Trait emotional intelligence is one of two major approaches to the conceptualization of EI. It refers to a group of personality-driven predispositions located at the lower level of major personality axes relating to adaptation—specifically, flexibility versus rigidity [38]. These predispositions or traits relate to mood and emotion regulation, self-control, sociability, and adaptability. Trait EI is not merely one’s perception of one’s own emotional ability but a ‘default reaction pattern’ for self-management in emotional and interpersonal settings. It is highly associated with well-being, personal and interpersonal functioning, and a broad range of health indicators. It serves as a protective factor against a range of detrimental physical and mental health outcomes and conditions [39]. We therefore chose trait EI as a factor to be associated with distress in our proposed model.

### 2.2. Social Level Resources: Social Support

A wealth of social-psychological research and theory has shown how powerful the presence of others is when it comes to individual outcomes [40]. Social support is perhaps the most important factor associated with effective coping in a broad range of settings, from war and natural disasters to health challenges [41,42,43], including the COVID-19 pandemic [14,18]. Social support refers to the extent to which individuals perceive others to be available to fulfil sharing, safety, and help needs [44]. It includes various aspects of the availability of others, ranging from ‘being there for someone’ i.e., providing psychological and emotional support and comfort, to instrumental availability, i.e., providing help in practical aspects of daily living. This type of support was of special interest to us because the COVID-19 outbreak confined many to their homes in lockdown. Studies conducted throughout the pandemic revealed how challenging loneliness can be [45], while also highlighting displays of empathy, compassion, and prosocial behavior associated with coping with the pandemic and the restrictions it imposed [46,47].

A study of students during the pandemic observed a significant effect of perceived family (social) and institutional support on mental health [27]. Another study found such support was negatively correlated with students’ perceived stress [26]. Furthermore, students with low perceived social support were more likely than those with high perceived social support to report anxiety and depressive symptoms [14]. Conversely, feelings of integration and good quality social bonds were associated with a lower likelihood of reporting impaired mental health during the pandemic [18].

### 2.3. Psychological Distress: Stress, Anxiety, and Depression

The human reaction to threat and crisis is well documented. Both our physical and our psychological reactions to threat are aimed at mobilizing resources to cope with a threat more effectively at the expense of less vital systems and processes. While such reaction patterns are functional, individuals and groups pay a steep price when threat conditions linger, posing a risk of exhaustion, burnout, and a range of pathologies. We focused on three concepts representing three points on the threat reaction continuum: stress, anxiety, and depression, broadly agreed to be components of the concept of ‘distress’ [48]. In the context of COVID-19, stress, depression, and anxiety were found to be more prevalent in females than males, and more common more in freshmen than sophomore students [49].

These three mental health conditions share some common symptoms. Although they are correlated, however, their core conceptualizations and documented manifestation and prevalence vary dramatically [50].

**Stress**. Psychological stress is a term describing a multi-systemic reaction pattern typical of a state in which individuals or groups identify a threat or an opportunity requiring a reaction. The reaction pattern is characterized by resource mobilization (e.g., sympathetic reaction patterns) typically prioritizing a ‘fight or flight’ reaction [51]. While stress can lead to adaptive behavior (such as motivational behavior, competition etc.), when left unattended for a long period, it may undermine physiological and cognitive functioning and take a heavy toll on individuals [52]. The stressed individual experiences emotional and physical tension, irritability, restlessness, and impatience.

According to evidence drawn from student samples the world over, student life can be very stressful. Students experience numerous challenges and try to cope with growing responsibilities and requirements in such areas as studying, personal, family, social, and sometimes professional life [53]. Many students are away from their families and their original support systems, in many cases for the first time [54]. Thus, although not often thought of this way, students may be defined as a vulnerable population (e.g., [10,11,55,56,57]). This suggests the value of our focus on this population [58].

**Anxiety.** Anxiety can be defined as a multi-level response pattern resulting from prolonged exposure to stress [59]. Anxiety is one of the most pivotal components of a broad range of psychological disorders and thus is a central component in the makeup of distress, as defined by numerous authors. Anxiety has been associated with a broad range of negative health, mental health, and functional outcomes (e.g., academic-related, work-related, etc.) [60]. Previous studies found individuals with anxiety struggle to adopt strategies that may support management or change their emotional states due to low emotional clarity, inability to process emotions, and deficient emotional regulation [61]. This, in turn, may imply difficulties in managing social relationships. A pioneer study by Schachter and Singer [62] concluded the arousal of any strong emotion tends to create the need to compare this reaction with others and, hence, produces a need for social affiliation. Yet Sarnoff and Zimbardo [63] showed that as fear increases, so too does the desire to affiliate with others, while the opposite is true for anxiety. 

**Depression.** Depression may be viewed as a general diagnosis reflecting a severe lowering of energy and motivation and receding cognitive, social-interpersonal, and daily function associated with negative mood and recurring negative thoughts [64]. Depressive symptoms include significant weight or appetite loss, sleep disturbance, psychomotor changes, loss of energy or fatigue, feelings of worthlessness (low self-esteem, feelings of guilt), hopelessness, diminished cognitive function, lower concentration, recurrent negative thoughts, and more. While depression as a diagnosed disorder causes a substantial amount of distress, in our context, depressive symptoms might be part of the components of distress even if they do not reach the severity or longevity criteria set by the diagnostic manual [65].

Depressive symptoms may be associated with distress through maladaptive emotion regulation strategies, such as rumination, self-blame, and avoidance [66]. The inability to regulate emotional responses in response to challenging circumstances (such as the threat of COVID-19) for a long period causes high distress usually linked to depression [67]. By the same token, depressive episodes have been found to occur as a response to stressful life events that exceed the individual’s coping capacity or resilience [68].

### 2.4. The Study Model

**Figure 1:**
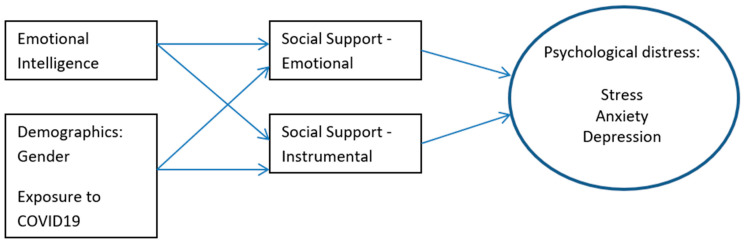
The Study Model.

Based on the literature review, we propose a model linking individuals’ pre-dispositions with social interactions to explain how people managed their distress reactions at the height of the COVID-19 crisis. Theoretically we would expect individual resources to work at the preliminary level of coping: background and demographic variables first and then individual resources: in our case, EI. We expected individual resources to help engage various types of support, to help mitigate the negative potential outcomes of threat and stress. Figure 1 summarizes this model.

The model proposes that after controlling for potential demographic intervening variables, such as gender and level of exposure, two types of social support (emotional and instrumental) will mediate the association between EI and the level of reported psychological distress due to the COVID-19 risk/crisis as expressed in stress, anxiety, and depression.

## 3. Method

### Setting and Sample

We conducted an online survey a week after the WHO declared the coronavirus outbreak a pandemic (17 March 2020). Three days before we sent out the survey, the Polish government introduced the first major restrictions related to social distancing and the covering of face and nose in public spaces. We recruited 159 students from the pedagogy department of a mid-sized Polish university to this study. Ninety-five percent of the participants were women. The mean age was 21.00 (sd = 3.39). The overwhelming majority (98%) reported being made aware of the COVID-19 risk only through the media. Actual statistical power calculated for this sample size, given an alpha level of 0.05 and considering the number of factors included in the model, was 0.96, substantially above the 0.80 standard set in the methodological literature.

## 4. Measures

### 4.1. Emotional Intelligence

EI was assessed using the TEIQue-SF [36], a 30-item version of the original questionnaire used to assess various aspects of trait EI. We used the total score of the questionnaire as our main variable. The measure maintains an acceptable construct validity and reliability, ranging from 0.67 to 0.88 [69].

### 4.2. Social Support

Social support was assessed with the Berlin Social Support scale [70], using two subscales of the original six, for a total of eight items. We used the available support subscale, assessing individuals’ perception of the psychological and emotional support available to them, and the provided support scale, representing the support individuals get on a practical daily level (instrumental support). The construct validity reported for this scale across cultures and languages is satisfying, and internal reliability ranges from 0.75 to 0.96 [71].

### 4.3. Psychological Distress

Psychological distress was assessed using three different concepts covering the continuum between immediate reaction to risk (perceived stress), emotional interpretation (anxiety), and an extreme outcome (depression).

**Perceived Stress.** Perceived stress was assessed using a single item question measuring a self-reported stress level (during the pandemic) on a 5 point-Likert scale, ranging from 1 = very low to 5 = very high. Numerous studies report high validity of a single measure of stress levels (e.g., [72]).

**Anxiety**. Anxiety was assessed using the anxiety dimension of the Brief Symptom Inventory (BSI) [73]. It includes six items related to self-reported clinically relevant psychological symptoms, such as nervousness or shakiness, tension, fear, feeling restless or scared. The general scale shows good internal consistency and reliability, ranging from 0.71 to 0.85.

**Depression.** We assessed depression using six items of the depression dimension from the Brief Symptom Inventory (BSI) [73]. The subscale measures the intensity of symptoms related to feelings of loneliness, hopelessness, losing interest in daily life, or suicidal thoughts. The general scale has good internal consistency reliability, ranging from 0.71 to 0.85.

**Demographics.** We gathered demographic information using a dedicated questionnaire. Individuals reported their gender, age, and level of exposure to the COVID-19 risk (ranging from none at all, to exposure only through the media, to being infected).

### 4.4. Procedure and Ethics

An online survey was designed on a platform with a direct link providing access for participants. The link was distributed electronically among students of the pedagogy department of a large university in southeastern Poland. The participants were assured of the anonymity of their responses, the voluntary nature of the study, and their right to withdraw at any point. We placed information about the study on the internal platform of the pedagogy department. Out of 300 students, 159 completed the survey. The study was conducted according to the ethics guidelines set out by the Ethics Committee of the university.

### 4.5. Data Analysis

After running basic descriptive statistics for the study variables, we tested the proposed model using path analysis in AMOS 20.0 (IBM, 2019). The path analysis yielded goodness of fit indices, as well as modification indices that guided the model modifications.

## 5. Results

### 5.1. Descriptive Statistics

We first examined the distribution and basic inter-correlations of the main variables. These are summarized in Table 1.

The preliminary descriptive statistics revealed adequate distributions with no expressed ceiling or floor effects and reasonable dispersion. The zero order Pearson’s correlations provided general support for our hypothesized model.

### 5.2. Model Testing

**Figure 2:**
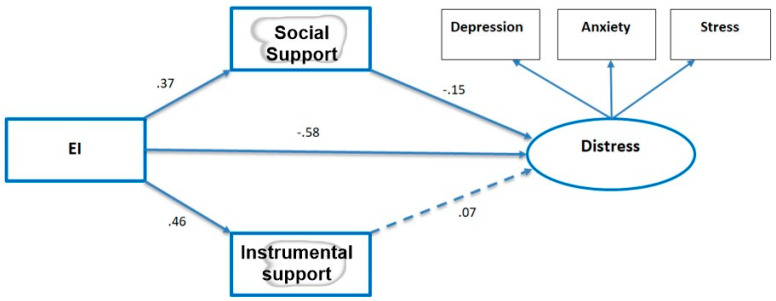
Empirical SEM Model (n = 159). Notes: Error terms have been deleted for the sake of clarity of presentation. All paths marked in solid lines are statistically significant at *p* < 0.04 or better. Component loadings on the latent distress factor: Stress = 0.70, Anxiety = 0.86, Depression = 0.84. Goodness of fit indices for the empirical model: Chi-square = 17.50, df = 6, *p* < 0.05. NFI = 0.95 CFI = 0.97, RMSEA = 0.06. Indirect effect of EI on distress = −0.06; 95% CI = −0.08/−0.04. Total effects of EI on distress = −0.64 (r square = 0.41).

We tested our model using SEM analysis in Amos 20.00. The results are summarized in Figure 2.

When we ran our hypothesized model, we did not receive appropriate fit to the data. We modified the model by erasing variables that showed no significant paths, namely demographics and the level of perceived exposure to COVID-19. We added a direct link between EI and the distress factor and correlated the two types of social support (as these emerged from the descriptive correlations). The modified empirical model showed very good fit to the data (see Figure 2). The model suggested psychological social support mediated the link between EI and distress in the hypothesized manner; i.e., EI was positively associated with social support which, in turn, was negatively associated with the latent distress factor. However, instrumental support did not show this association. The hypothesized structure of the latent distress factor was supported (see Figure 2 and Table 1).

To further validate the results we also ran a moderation analysis to test an alternative model in which social support moderates the association between EI and distress. We used PROCESS moderation model (Model 1) analysis in SPSS [74]). While the main effect of EI on distress was maintained, the moderation effect was not statistically significant for either of the two measures of social support. Table 2 summarizes the results of this test.

Our results provided support for the mediation model.

## 6. Discussion

The COVID-19 pandemic took and still takes a heavy psychological toll on individuals and groups (e.g., [74]). This study tested a model of individual and social resources that may buffer the detrimental effect of exposure to the COVID-19 risk among young college students. We used perceived stress, anxiety, and depression, often mentioned in the literature as components of psychological distress, as our outcome variables. Our proposed model suggested that the effect of demographic characteristics and EI on distress may be mediated by social support. In other words, EI works to alleviate distress by recruiting and making the most of social support—which is broadly known to buffer the effects of threats and stressors in a wide range of settings. This is in addition to its direct effect on distress.

Our results, while generally supportive of this model, showed that social support had a differential effect on distress. We used two subscales of social support: one referred to emotional social support, such as offering a supportive shoulder, a trusting relationship, and an attentive ear; the second referred to more practical support, such as help with everyday chores. We found both subscales were associated with EI, but only emotional support was negatively associated with distress while practical support was not associated with it. These results may strengthen our understanding of how personal and social resources may be recruited to ameliorate the negative effects of exposure to threat. They suggest that in our sample, personal-emotional resources served to recruit interpersonal support resources (especially emotional ones) and these, in turn, were negatively associated with our outcome variables representing distress.

Another interesting finding was that none of the demographic characteristics entered the model. One possible explanation is the relatively homogenous sample in terms of gender and age. Another potential explanation is that EI masked the known effects of gender and age, for example, on the expression of distress, and this may attest to the relevance and power of the concept of EI in such settings. These results offer unique insights into the way young, healthy adults mobilize personal resources to cope with a real-life threat in ‘real time’. They accord with numerous studies showing how individuals and groups cope with stress using personal resources, especially EI (e.g., [5,75]). Our results also add to our understanding of the relationships between EI and stress outcomes beyond mere association: they draw a line from the psychological potentials indicated by the concept of EI through the recruitment and utilization of social support to alleviate the effects of a threat. This ‘line’ clarifies how personal resources translate into actions in the interpersonal realm to affect experience in times of crisis or threat.

An alternative approach to the analysis of our data could have adopted a moderation model: does social support moderate the effect of EI on distress? The literature includes competing mediation and moderation models in the exploration of the associations between EI and social support vis-à-vis measures of wellbeing or distress. Thus, for example, a study has found that the interaction between measures of EI and social support yielded a significant result [75]. In that study the interaction found between EI and social support was interpreted as one in which individuals high in EI did not show a need for social support, while for individuals with lower EI, social support becomes a crucial resource for emotional outcomes. A moderation effect in our context could mean that in the presence of social support, EI’s association with distress is dramatically weaker. So—does social support make EI redundant? From the existing literature on both EI and social support we know that EI is strongly associated with interpersonal dynamics, and interpersonal communication which are pivotal in fostering and maintaining social relations and support [37,38,42]. Thus, in our context, the mediation model makes a better fit to what we know of the nature of EI and social support. In our sample, the results indeed supported the mediation model. Our results echo those of Zeidner and Matthews [76] who found a similar mediation effect using different measures for the same concepts, in a different sample.

Thus it is also worthwhile to briefly discuss the potential meaning of the association between EI and social support, as it may reflect one of EI’s most important functions—one’s ability or tendency to interact effectively with others, recruiting relationships and interpersonal relations as means of attaining a goal. In our case, this means recruiting others and utilizing relationships to attain support, especially (so it seems) social and emotional support. While the theoretical literature on EI mentions the ’manipulative’ nature of the concept in interpersonal settings, this study exemplifies how EI helps use relationships to get support. Manipulation in this context is not necessarily negative or exploitive, but rather more goal oriented in nature.

Our results add to the knowledge of links between stress and health during a pandemic and point to the pivotal role of social support, especially emotional support. Other studies have similarly found that communicating with families and friends helped individuals deal with stress and anxiety during COVID-19 [10].

### The Study’s Contribution

Beyond supporting the proposed model, our results may also shed new light on aspects of mobilizing personal and interpersonal resources in times of crisis, at least in a student population: dealing with young adults, coping with new challenges on the academic, personal, and interpersonal levels, students make an excellent case study for exploring resource mobilization during emerging adulthood: psycho-social theory emphasizes interpersonal relationships, asserting and forming self-identity, and intimate relationships as major developmental axes in this period [77]. This could account for the importance of social-emotional support in our model: emotional support reflected, for instance, in expressing empathy [78], may be a prerequisite of distress alleviation, thus illustrating a basic need of human development—the need for belonging and affiliation. Simply stated, our participants needed some (emotional and social) help from their friends or family members.

Additional added value stems from our findings supporting the path from individual resources through social support to psychological outcomes under stress: EI has been extensively studied and a robust body of evidence links it to better psychological and often—physical outcomes. However—less is known on how EI works to alleviate distress. Our results suggest at least one possible path through which it may help shape emotional outcomes under stress, thus helping us broaden our understanding of the concept and its dynamics in this context.

## 7. Study Limitations

The study had some limitations. First, we had an adequate size sample, but it was drawn from a single university in a single city in Poland. This might limit our ability to generalize our results beyond a specific location and culture, although results of other studies suggest our outcomes are applicable to numerous similar settings. Second, we used self-report measures because we lacked direct access to participants during the lockdown. While we used well documented and validated measures, the use of self-report measures has challenges and issues (e.g., [79]). Third, our study design permitted intricate analyses but did not allow causal inference. Future studies may use more diverse settings and populations, utilize behavioral measures alongside self-report measures, and perhaps adopt longitudinal study designs to better examine sequential and causal associations.

## 8. Future Work

Cumulative data and results may help us monitor and examine issues in real time, as the COVID-19 crisis continues to unfold, including how well-being and distress are experienced and managed in varying populations. Despite hundreds of studies on the psychological problems of the COVID-19 pandemic, many aspects are still unclear, and more research is needed on how remote learning, academic workload, separation from peers and university, health problems, including among loved ones, and fears of contagion (or wearing protective masks, e.g., [80]) may be detrimental to health.

Future work could provide a more in-depth examination of the relationship between the various ways of coping with COVID-19 as a stressor and their consequences for students’ physical and mental health and academic achievement, as the effects of the pandemic may continue for a long time. It would also be interesting to explore whether people who were able to seek, build, and receive social support, especially of an emotional nature, were able to cope with the effects of the pandemic on physical and mental health and even achieve post-traumatic growth (e.g., [81]). Better understanding of the underlying dynamics will help us to inoculate populations and groups against the physical threat of infection and also to inoculate them, so to speak, against the negative psychological and social consequences of the pandemic.

## 9. Conclusions

Existing evidence links the concepts of EI and social support with various daily outcomes, chief of which are wellbeing and distress. While each is well documented in various settings, the way EI and social support interact and work to shape these outcomes is less well known. This study tested a proposed model in which EI is seen as a personal-level resource that works to manage and regulate emotions and, at the same time, to recruit social support (especially emotional social support) which, in turn, is associated with distress. The model was tested in a sample of students at a university in southeastern Poland, at the height of the COVID-19 outbreak—a global crisis associated with increased levels of distress in diverse populations. Our results support the hypothesized model and suggest different routes by which EI works to help us buffer difficult experiences and challenges. At the same time, the results highlight the importance of emotional social support in times of crisis, above and beyond the instrumental added value of social support.

## Figures and Tables

**Table 1:** Descriptive Statistics and Zero-Order Correlations among Main Study Variables (n = 159).

	Mean SD	Reliability	1	2	3	4	5	6	7	8
1. Gender	95% F 5% M	–	–							
2. Exposure		–	0.03	–						
3. EI	132.63 17.46	0.79	0.09	0.06	–					
4. Psych sup	13.70 2.34	0.84	−0.08	0.02	0.36 **	–				
5. Inst sup	14.00 2.48	0.91	−0.07	0.09	0.45 **	0.81 **	–			
6. Stress	3.42 0.98	–	−0.09	−0.11	−0.31 **	−0.08	−0.23 **	–		
7. Anxiety	9.49 6.12	0.91	0.04	−0.05	−0.39 **	−0.25 **	−0.24 **	0.42 **	–	
8. Depression	8.27 5.72	0.89	0.04	−0.09	−0.55 **	−0.25 **	−0.27 **	0.39 **	0.63 **	–

** *p* < 0.01.

**Table 2:** A summary of PROCESS mediation analysis for social support (two separate measures) (n = 159).

	Coefficient	SE	t	*p*
Constant	21.92	12.63	1.73	0.08
EI	−0.09	0.09	−0.96	0.33
Emotional support	−0.06	0.91	−0.06	0.94
Moderation model	−0.01	0.00	−0.09	0.92
R square = 0.28, F = 20.78 (df = 3), *p* < 0.00				
	**Coefficient**	**SE**	**t**	***p***
Constant	19.33	10.17	1.90	0.05
EI	−0.08	−0.08	−0.99	0.32
Emotional support	0.14	.072	0.19	0.84
Moderation model	−0.01	0.00	−0.31	0.75
R square = 0.28, F = 20.33 (df = 3), *p* < 0.00				

## Data Availability

Data can be made partially available upon request from the 3rd author.
